# Recovery after Exercise-Induced Muscle Damage in Subjects Following a Vegetarian or Mixed Diet

**DOI:** 10.3390/nu16162711

**Published:** 2024-08-15

**Authors:** Nicole Presti, Todd C. Rideout, Jennifer L. Temple, Brian Bratta, David Hostler

**Affiliations:** 1 Center for Research and Education in Special Environments, Exercise and Nutrition Department, University at Buffalo, Buffalo, NY 14214, USA; npresti@buffalo.edu; 2 Exercise and Nutrition Department, University at Buffalo, Buffalo, NY 14214, USA; rideout@buffalo.edu (T.C.R.); jltemple@buffalo.edu (J.L.T.); 3 Athletics Department, University at Buffalo, Buffalo, NY 14214, USA; bbratta@buffalo.edu

**Keywords:** plant-based diet, omnivore diet, isometric strength, concentric strength, performance nutrition, sports nutrition, protein

## Abstract

It is unclear if following a vegetarian diet affects muscle recovery after exercise-induced muscle damage (EIMD). Sixteen vegetarians (VEG) and sixteen mixed dieters (MIX) performed a vertical jump, quadriceps femoris maximal isometric, and isokinetic concentric strength tests prior to and five days following the EIMD protocol. The quadriceps muscle was injured by performing eccentric contractions. Diet: MIX consumed more g/kg of animal protein (*p* < 0.001) and EAA (*p* < 0.05) except for isoleucine. VEG consumed more plant protein (*p* = 0.001). Isometric strength: MIX recovered post-day 2, VEG recovered post-day 4 (group (*p* = 0.07), time (*p* < 0.001)). Concentric contractions at 60 degrees per second: Both recovered post-day 1 (group (*p* = 0.27), time (*p* = 0.05)); 180 degrees per second: MIX recovered post-day 2, VEG recovered post-day 5 (group (*p* = 0.10), time (*p* < 0.001)); and 240 degrees per second: MIX recovered post-day 1, VEG did not recover by post-day 5 (group (*p* = 0.01), time (*p* < 0.001)). Vertical jump: Both recovered post-day 3 (group (*p* = 0.45), time (*p* < 0.001)). MIX recovered isometric strength 2 days faster, concentric strength was up to 5 days faster, and soreness was 1–4 days faster when compared to VEG. Both groups had similar recovery time for power.

## 1. Introduction

Exercise-induced muscle damage (EIMD) occurs after an unaccustomed exercise is performed. Signs and symptoms include muscle soreness, stiffness, tenderness, pain, swelling, and oxidative stress [1,2]. EIMD decreases muscle function which impairs strength, power, speed, and range of motion [2]. Side effects can be experienced immediately after exercise and last for several days [3,4]. Previous studies have shown how branched-chained and essential amino acids can shorten the duration of symptoms of EIMD [5,6,7]. With a vegetarian diet typically containing less protein and having lower protein bioavailability than a mixed diet, it is unclear how this affects recovery [8,9].

Resistance training is known to stimulate muscle protein synthesis (MPS). However, in a fasted state, the rate of muscle protein breakdown (MPB) will exceed the rate of MPS. When amino acids are consumed, MPS can exceed MPB [10]. While MPS is associated with hypertrophy, it also aids in the remodeling of contractile and structural proteins [11]. MPS could be affected by a vegetarian diet due to the lower protein bioavailability and lower protein intake that are typically seen in vegetarian diets [8,9].

The branched-chain amino acid (BCAA) leucine is of most concern because it has the strongest anabolic properties. Leucine stimulates MPS by activating mammalian target of rapamycin (mTOR) [12,13]. Due to lower protein availability, athletes consuming a vegetarian diet need to consume more protein compared to athletes who consume a mixed diet to meet their leucine recommendation. For example, there is 2.7 g of leucine in 25 g of whey protein. For an athlete consuming plant protein, they would need to consume 38 g of pea, 40 g of soy, or 45 g of wheat protein to consume 2.7 g of leucine [14,15].

In addition to remodeling proteins, BCAAs reduce muscle soreness and inflammation after EIMD. A study completed by Matsumoto et al. showed a decrease in muscle soreness and inflammation after EIMD when subjects consumed a BCAA drink when compared to consuming the placebo [5].

Plant-based diets can be beneficial for athletic performance. They are typically higher in carbohydrates, which provide athletes with energy, and antioxidants, which reduce oxidative stress [16,17,18,19]. Disadvantages of following a plant-based diet include lower protein bioavailability and lower creatine intake, which negatively affect muscle growth [8,15,20,21]. While there are studies investigating how plant-based diets affect athletic performance, less is known with respect to muscle recovery [16,19,22,23,24]. One study completed by Leach investigated the effects a vegan diet has on muscle recovery after a bout of exercise. Recovery was determined by tracking EIMD biomarkers in the blood 0, 1, 3, 24, 48, and 72 h after completing a muscle damage protocol. Results showed that a vegan diet had a protective effect after subjects performed an EIMD protocol when it came to levels of creatine kinase and interleukin 1-beta but we did not see this effect with c-reactive protein, tumor necrosis factor alpha, and interleukins 6 and 10 [25]. Thus, although a plant-based diet may offer some protection against muscle damage, the current protein recommendations for athletes are derived from research conducted in people who consume meat, with no consideration of vegetarian or vegan diets. Therefore, it is important to further explore the effects of a plant-based diet on muscle recovery. The purpose of this study was to investigate the difference in the recovery of strength and power and to assess soreness after a bout of exercise causing EIMD in people who follow a plant-based diet versus people who follow a mixed diet. We hypothesized that people who follow a mixed diet will have faster recovery of strength, power, and soreness when compared to people who follow a plant-based diet.

## 2. Materials and Methods

### 2.1. Subjects

Forty-two subjects were recruited for this study. They were invited to participate if they were between the ages of 18 and 39, did not have neuromuscular disease or hypertension, and had no lower leg injuries in the past year. Subjects could not be pregnant, lactating, trying to become pregnant, use tobacco, or have contraindications to exercise. Subjects had to have previously followed (minimum of three months) and continued to follow a mixed or plant-based diet. To be placed in the plant-based diet group, subjects could consume eggs and dairy but had to exclude red meat, poultry, pork, and seafood. Subjects were instructed to follow their typical diet for the duration of the study.

During visit 1, a consent and Physical Activity Readiness Questionnaire for Everyone (PAR-Q+) form was completed in addition to confirming that they met the inclusion criteria and reporting their demographics. Subjects were educated by a registered dietitian on how to fill out a 3-day diet recall consisting of one weekday, one weekend, and one weekday or weekend. The subjects had the duration of the study (2 weeks) to complete the diet recall. Diet recalls were analyzed with the NUTRITION DATA SYSTEM FOR RESEARCH (NDSR) Database (Version 2023 © 2023 Regents of the University of Minnesota).

**Figure 1:**
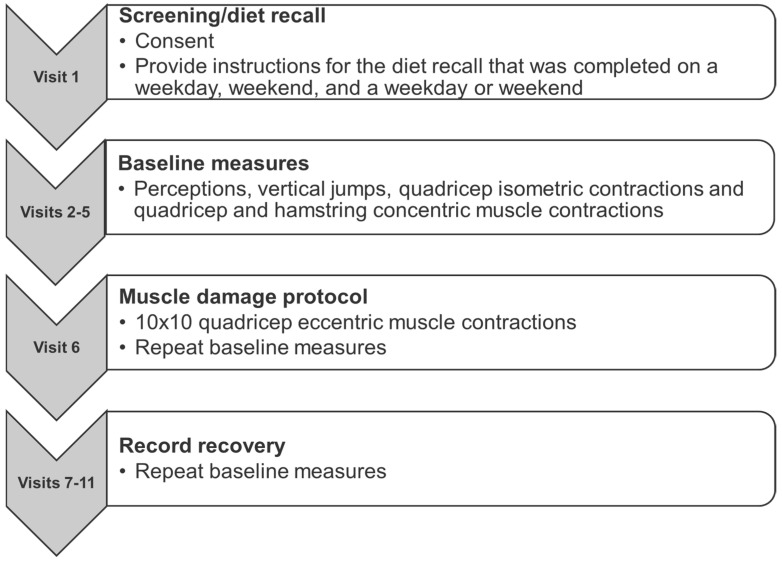
Methods.

There were 11 visits in total. During visit 2, height, mass, body mass index (BMI), and resting vitals (heart rate and blood pressure) were recorded. Baseline measurements were performed on their dominant leg during visits 2–5 to familiarize subjects with the protocol and ensure maximal effort was recorded (Figure 1).

### 2.2. Baseline Measurements

#### 2.2.1. Soreness

A 10 cm visual analog scale (VAS) and pressure algometer were used to rate soreness. Subjects were instructed to rate their pain while standing, sitting, and walking by marking a line on a 10 cm scale, with one end of the scale being no pain and the other end being pain as bad as it possibly could be. The pressure algometer recorded the pressure pain threshold while sitting and standing. The pressure algometer gradually applied pressure to the quadriceps. Subjects were instructed to tell the study team member when pressure became pain by saying “now”, and the pressure was withdrawn and recorded.

#### 2.2.2. Power

Jump height was measured by performing a vertical jump on a Just Jump System, Probotics, Inc., Huntsville, AL, USA. (jump mat). Subjects performed a maximal effort countermovement vertical jump by standing on the jump mat with their feet shoulders width apart, placing their hands on their hips, squatting down, and jumping vertically at maximal effort. The highest jump out of three attempts was recorded.

### 2.3. Isometric Strength

Subjects performed a knee extensor force test on a Biodex Medical Systems, Rev 4.63 10 May 2018, Shirley, NY, USA (Biodex dynamometer). Tests were conducted by the same researcher, whose coefficient of variation was previously determined to be under 5%. Three maximal isometric contractions of the knee extensor (at 90 degrees of knee flexion) were completed for six seconds. There was a 2 min rest between contractions. The highest torque was recorded.

### 2.4. Concentric Contractions

Subjects performed a concentric torque test on the Biodex dynamometer. Subjects completed five maximal concentric contractions of the knee flexor at 60, 180, and 240 degrees per second. The highest torque of the three attempts was recorded.

### 2.5. Muscle Damage Protocol

The muscle damage protocol was scheduled no later than one week after visit 2. The muscle damage protocol was a series of eccentric muscle contractions completed on the Biodex dynamometer. Subjects performed 10 sets of 10 contractions with 30 s of rest between each set. Baseline measurements were repeated after the muscle damage protocol. Subjects came to the lab for 5 consecutive days post-testing scheduled 22–24 h apart. During each of these visits, subjects repeated baseline measurements.

### 2.6. Statistical Analysis

An unpaired *t*-test was used to analyze demographics. Two-way ANCOVA was used to analyze study outcomes while accounting for group differences in height, mass, and BMI. In order to comply with assumptions of a paired *t*-test and a two-way ANCOVA, the data were tested for normality. If the data were not normally distributed, a log transformation was performed, and data were rechecked for normality. A log transformation was applied to concentric strength at 60 degrees per second; fat, protein, and plant protein in grams; age BMI; isoleucine, threonine, leucine, lysine, and methionine in grams; and all the essential amino acids in g/kg except for tryptophan for the purpose of establishing normality. For data that could not be transformed, animal protein in grams and animal and plant protein in g/kg, a nonparametric test was conducted.

To account for subject variability, isometric strength, concentric strength, and the vertical jump are expressed as percent change from baseline. When appropriate, ROUT (Q = 1%) outliers were removed. Outliers were removed for fat, protein, animal protein, and all essential amino acids in grams; fat, protein, animal protein, and methionine in g/kg; and concentric strength at 60 degrees. Data were analyzed with GraphPad Prism 10 and Statistical Package for the Social Sciences 29 (SPSS). Significance was set at *p* ≤ 0.05.

## 3. Results

### 3.1. Demographics

The power calculation was created by G*Power 3.1.9.7. Based on previous studies of muscle damage measured by isokinetic dynamometry, six subjects per group are sufficient to identify differences in the primary outcomes (a = 0.05, b = 0.90). Due to a high influx of subjects, we ran 16 subjects per group to improve statistical power. Forty-two subjects met the criteria and agreed to take part in this study. Nine subjects dropped out due to scheduling conflicts. One subject finished but was not included in the analysis because we could not confirm if he consumed meat or not. Therefore, thirty-two subjects completed the study. Height (*p* = 0.001), weight (*p* = 0.002), and BMI (*p* = 0.02) were higher in the MIX group when compared to the VEG group. There was no difference in age between the two groups (*p* = 0.23) (Table 1).

### 3.2. Diet

Nutrient intake is provided in Table 2 and Table 3. The MIX group had a higher intake of calories (*p* = 0.006) and consumed more grams of carbohydrates (*p* = 0.02), total protein (*p* < 0.001), and animal protein (*p* < 0.001). The VEG group consumed more plant protein (*p* = 0.02). Both groups consumed a similar amount of fat (*p* = 0.10). When taking weight into consideration, there was no difference between groups for kcals/kg/day (*p* = 0.66) or g/kg/day of carbohydrates (*p* = 0.15), protein (*p* = 0.13), or fat (*p* = 0.91). The VEG group consumed more g/kg/day of plant protein (*p* = 0.001). The MIX group consumed more g/kg/day of animal protein (*p* < 0.001).

When examining essential amino acid (EAA) consumption, MIX consumed more grams of all the EAAs (*p* < 0.002). MIX consumed more g/kg/day of all the EAAs (*p* < 0.002) except for isoleucine. The VEG group consumed more isoleucine (*p* < 0.001).

### 3.3. Strength: Isometric

**Figure 2:**
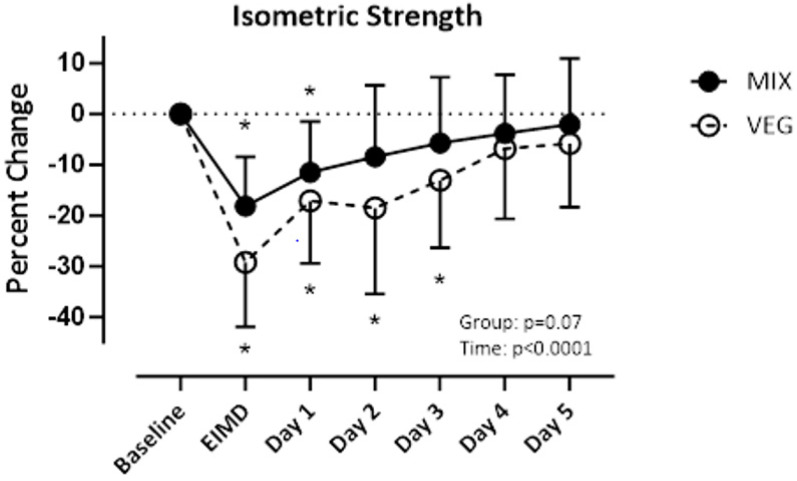
Percent change of quadricep isometric strength from baseline. Significance set at *p* < 0.05. VEG = vegetarian group; MIX = mixed diet group; EIMD = day of the exercise induced muscle damage protocol. * = different from baseline.

ANCOVA results revealed mass (*p* = 0.92), height (*p* = 0.28), and BMI (*p* = 0.114) did not influence isometric strength. There was a difference over time (_F(3680, 110.4_) = 27.36; *p* < 0.001) but not between groups (_F(1,30)_ = 3.446; *p* = 0.07) for change in isometric strength following EIMD. The VEG group recovered isometric strength by post-day 4 and the MIX group recovered by post-day 2 (Figure 2).

### 3.4. Strength: Concentric

**Figure 3:**
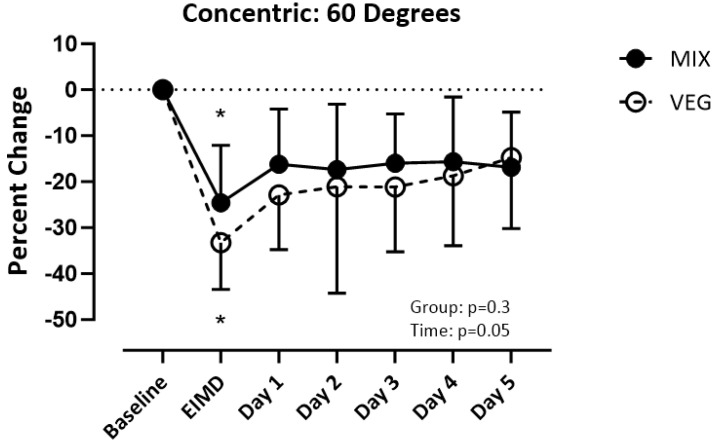
Percent change of quadricep concentric strength at 60 degrees per second from baseline. Significance set at *p* < 0.05. VEG = vegetarian group. MlX = mixed diet group. ElMD = day of the exercise induced muscle damage protocol. * = different from baseline.

**Figure 4:**
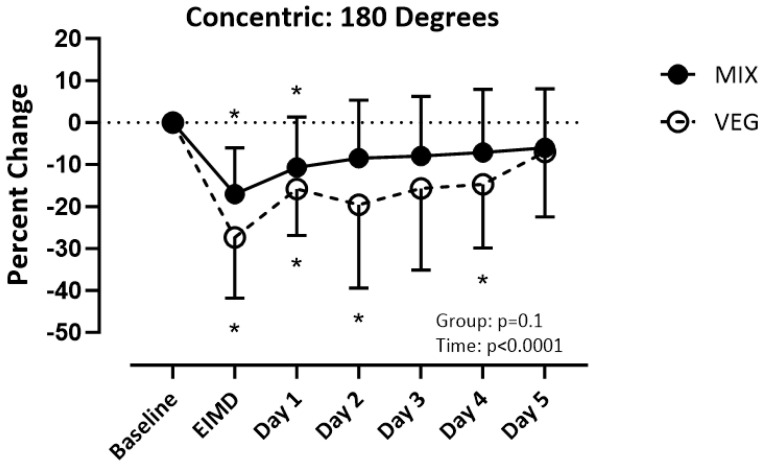
Percent change of quadricep concentric strength at 180 degrees per second from baseline. Significance set at *p* < 0.05, VEG = vegetarian group. MlX = mixed diet group. ElMD = day of the exercise induced muscle damage protocol. * = different from baseline.

**Figure 5:**
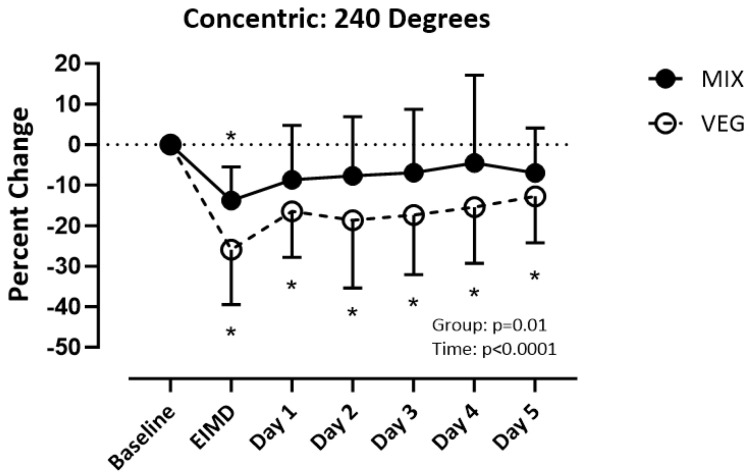
Percent change of quadricep concentric strength at 240 degrees per second from baseline. Significance set at *p* < 0.05. VEG = vegetarian group. MlX = mixed diet group. ElMD = day of the exercise induced muscle damage protocol. * = different from baseline.

ANCOVA results revealed mass (*p* > 0.4), height (*p* = 0.5), and BMI (*p* > 0.3) did not influence the results for concentric strength at 60, 180, and 240 degrees per second. There was a difference over time (_F(1.540, 45.94)_) = 3.469; *p* = 0.05) but not between groups (_F(1,30)_) = 1.248; *p* = 0.27) for concentric strength of the quadriceps at 60 degrees per second. Both groups recovered by post-day 1 (Figure 3). There was a difference over time (_F (3.372, 101.1)_ = 14.75; *p* < 0.001) but not between groups (_F(1,30)_ = 2.887; *p* = 0.10) for concentric strength at 180 degrees per second. The VEG group recovered by post-day 5 and the MIX group recovered by post-day 2 (Figure 4). There was a difference over time _(F(3.243, 97.30)_ =10.12; *p* < 0.001) and between groups (_F(1,30)_ =7.029; *p* = 0.01) for concentric strength at 240 degrees per second. The MIX group had a faster recovery time recovering on post-day 1, while the VEG group did not recover by-post day 5 (Figure 5).

### 3.5. Vertical Jump

**Figure 6:**
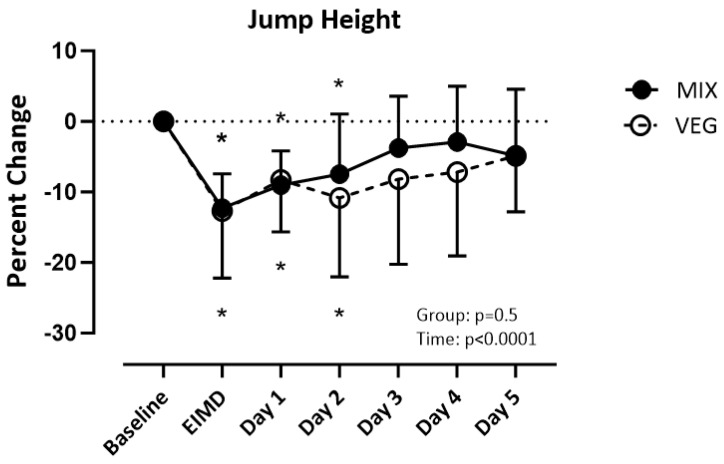
Percent change of jump height from baseline. Significance set at *p* < 0.05. VEG = vegetarian group. MlX = mixed diet group. ElMD = day of the exercise induced muscle damage protocol. * = different from baseline.

ANCOVA results revealed mass (*p* = 0.261), height (*p* = 0.115), and BMI (*p* = 0.299) did not influence jump height. There was a difference over time (_F(3.409, 102.3)_ = 15.22; *p* < 0.001) but not between groups (_F(1,30)_ =0.5760; *p* = 0.45) for the vertical jump. Both groups recovered jump height by post-day 3 (Figure 6).

### 3.6. Soreness

There was no difference over time (_F(3.943, 118.3)_ =1.958; *p* = 0.11) but there was a difference between groups (_F(1,30)_ =6.078; *p* = 0.02) when examining the pressure pain threshold while sitting. Both groups did not differ from the EIMD protocol by post-day 5. While standing, the pressure pain threshold was different over time (_F(3.746, 112.3)_ =2.621; *p* = 0.04) and between groups (_F(1,30)_ =4.452; *p* = 0.04). The VEG group differed from EIMD on post-day 3 only. The MIX group differed from the EIMD protocol by post-day 5.

The VAS was completed while walking, sitting, and standing. For walking, there was a difference over time (_F(2.467, 74.01)_ =19.26; *p* < 0.001) but not between groups (_F(1,30)_ =0.0148); *p* = 0.90). The VEG group recovered by post-day 5, while the MIX group recovered by post-day 2. When sitting, there was a difference over time (_F(1.768, 53.03)_ =10.04; *p* < 0.001) but not between groups (*p* = 0.24). The VEG group did not recover by post-day 5 and the MIX group recovered by post-day 4. When standing, there was a difference over time (_F(2.103, 63.10)_ =14.02; *p* < 0.001) but not between groups (_F(1, 30)_ =0.2595; *p* = 0.61). The VEG group did not recover by post-day 5 and the mixed group recovered by post-day 1.

## 4. Discussion

In this present study, we compared muscle recovery after performing an EIMD protocol in people who habitually follow a vegetarian diet to those who habitually follow a mixed diet. The MIX group recovered isometric strength 2 days quicker when compared to the VEG group. When investigating recovery of concentric strength, the MIX group recovered concentric strength faster as the speed increased. Although a similar recovery time was observed between the two groups when performing a concentric contraction at 60 degrees per second, the MIX group had a 3-day-faster recovery at 180 degrees per second and a 4-day-faster recovery at 240 degrees per second. While only the 240 degrees per second speed showed a statistically significant difference, in application, if an athlete can recover strength 2–4 days faster than their opponent, that would give them a competitive edge.

The MIX group recovered isometric strength two days faster than the VEG group. For athletes who play 2–3 games per week, being able to recover strength 2 days quicker would be of benefit [26]. One potential explanation for the results is overall protein intake. While g/kg/day of protein between both groups was not statistically significant, the lower end of protein recommendations for athletes is 1.2 g/kg/day, which the MIX group was just shy of consuming (1.1 g/kg/day) and the VEG group (0.9 g/kg/day) was further away from consuming. In addition, subjects in the VEG group consumed less leucine when compared to the MIX group. Since leucine is known to aide in the recovery process by increasing MPS, decreasing MPB, and suppressing exercise-induced muscle damage markers in the blood, it may explain why the MIX group recovered isometric strength two days faster [13,27]. A second explanation for this finding is the higher protein availability found in animal vs. plant-based protein. Ciuris et al. investigated overall protein absorbability using the Digestible Indispensable Amino Acid Score (DIAAS) in endurance athletes following a vegetarian or mixed diet. They reported that athletes who consumed a mixed diet had an 11% higher DIAAS and 43% more available protein per gram when compared to the vegetarian group. In application, a vegetarian athlete weighing 64 kg who aims to consume 1.2 g/kg/day would need to consume an extra 10 g of protein per day [8]. It is clear in our study that the MIX group consumed more animal protein when compared to the VEG group, suggesting higher bioavailability (however, we did not measure protein quality). In addition, the MIX group consumed more EAAs (except for isoleucine). The EAA leucine can improve muscle recovery after exercise, which may explain why the MIX group recovered isometric strength two days quicker than the VEG group [27,28].

For concentric contractions, both groups recovered by post-day 1 at the slower speed (60 degrees per second), but the mixed group recovered quicker at the faster speeds (180 and 240 degrees per second). While some studies report that protein can improve recovery of concentric strength after exercise [29,30], others do not [31,32]. Additional research investigating the role of protein quality in strength recovery after EIMD is warranted. Despite the improved recovery at higher concentric speeds, there were no differences in recovery between the groups for the vertical jump. Since muscle damage was only conducted on one leg, the other leg may have compensated for any decrements in the injured leg. Additionally, the subjects were not trained athletes and likely had not developed the motor patterns required for optimal jump height. Future studies investigating muscle recovery should investigate jump height using only the damaged leg in trained subjects.

During the soreness assessments, the MIX group recovered 1–4 days faster when compared to the VEG group while completing the VAS test. This may be due to the increased consumption (g/kg) of the BCAAs leucine and valine in the MIX group. This aligns with Matsumoto et al., who showed that the consumption of BCAAs suppresses inflammation and muscle damage after exercise [5]. One limitation to this study was that the pressure algometer maxed out at 22 pounds of force during the pain-upon-use test. Subjects from both groups maxed out the pressure algometer, which did not provide us this the most accurate reading. If a subject maxed out, their recorded pain-upon-use score was 22 across all 6 days, which did not allow us to see the number increase as subjects became less sore.

An additional limitation to consider is interindividual variability. While most of our subjects were untrained, a few were, which could have affected their course of recovery. Furthermore, some subjects required less coaching. During the vertical jump, some subjects were already familiar with the protocol and demonstrated max effort from day 1, while others were not, which could have affected the results. Lastly, pain threshold will vary between subjects. This study required maxed effort, and subjects with higher pain thresholds may provide greater effort than those with a lower pain threshold. To best account for subject variability, we expressed our results as a percent change from baseline.

Future research should continue to investigate the effects a vegetarian diet has on muscle recovery; therefore, proper protein recommendations can be made to vegetarian athletes. Future research should focus on using trained subjects from different avenues of sport (endurance, resistance, team, club, etc.), as their course of recovery may be different than untrained subjects. In addition, using a higher calibrated pressure algometer and only performing a vertical jump on the injured leg will lead to more accurate results.

Given the limited number of studies published on the topic of muscle recovery after EIMD in vegetarians, future research is warranted. Future research should continue to investigate the effects a vegetarian diet has on muscle recovery and how it affects muscle recovery in trained subjects, and it should use alternative assessments such as markers of muscle damage in the blood. This will allow sports dietitians to make appropriate protein recommendations for athletes following a plant-based diet.

## 5. Conclusions

In subjects following a mixed diet, recovery of isometric strength was 2 days faster, concentric strength was up to 5 days faster, and soreness was 1–4 days faster when compared to VEG. Both groups had similar recovery time for power. This may be due to lower protein bioavailability or lower protein intake. It is our recommendation that sport dietitians who are counseling athletes following a vegetarian diet should verify that they are consuming enough protein, and if not, work on increasing their protein intake through food or supplementation. In addition, sport dietitians can improve the bioavailability of plant protein by having their athletes consume complementary protein and using protein supplements such as pea or soy. 

## Figures and Tables

**Table 1:** Subject demographics.

	Total (*n* = 32)	VEG (*n* = 16)	MIX (*n* = 16)
Age (yr)	26.0 ± 4.0	24.6 ± 2.9	26.4 ± 4.9
Height (cm) *	170.3 ± 8.3	165.8 ± 7.5	174.8 ± 6.7
Weight (kg) *	73.5 ± 21.2	62.4 ± 14.8	84.6 ± 21
BMI (kg/m^2^) *	25.1 ± 6.4	22.6 ± 5.3	27.5 ± 6.6
Sex			
Male (*n*=)	14	4	10
Female (*n*=)	18	12	6
Race			
White (*n*=)	11	2	9
Asian (*n*=)	20	13	7
Black (*n*=)	1	1	0

* = different between groups.

**Table 2:** Macronutrient and essential amino acid intake in grams.

Macronutrients	Total (*n* = 32)	VEG (*n* = 16)	MIX (*n* = 16)	*p* Value
Energy (kcals) *	1789.7 ± 549.4	1532.8 ± 384.6	2046.6 ± 579	*p* = 0.006
Carbohydrates (g) *	240.1 ± 60.7	216.1 ± 40.9	264.1 ± 68.6	*p* = 0.02
Fat (g)	62.4 ± 27.8	55 ± 27.3	69.9 ± 27.1	*p* = 0.10
Total protein (g) *	73.4 ± 40.1	52.4 ± 13.4	94.4 ± 46.9	*p* < 0.001
Plant protein (g) *	34.9 ± 9.3	38.4 ± 9.4	31.5 ± 8.0	*p* = 0.02
Animal protein (g) *	38.5 ± 39.9	14 ± 13.5	62.9 ± 42.8	*p* < 0.001
Essential Amino Acids				
Isoleucine (g) *	3.3 ± 2.0	2.2 ± 0.8	4.3 ± 2.2	*p* = 0.001
Leucine (g) *	5.7 ± 3.2	4.0 ± 1.4	7.3 ± 3.7	*p* < 0.001
Valine (g) *	4.6 ± 5.1	4.4 ± 6.9	4.9 ± 2.3	*p* = 0.001
Threonine (g) *	2.8 ± 1.7	1.8 ± 0.6	3.7 ± 2.0	*p* < 0.001
Lysine (g) *	4.4 ± 3.3	2.6 ± 1.1	6.3 ± 3.8	*p* < 0.001
Methionine (g) *	2.2 ± 3.5	1.0 ± 0.4	3.4 ± 4.7	*p* = 0.001
Phenylalanine (g) *	3.4 ± 1.6	2.5 ± 0.7	4.2 ± 1.8	*p* = 0.001
Histidine (g) *	1.9 ± 1.1	1.3 ± 0.4	2.6 ± 1.3	*p* = 0.001
Tryptophan (g) *	0.9 ± 0.5	0.6 ± 0.2	1.1 ± 0.5	*p* = 0.001

* = different between groups.

**Table 3:** Macronutrient and essential amino acid intake in grams/kilogram.

Macronutrients	Total (*n* = 32)	VEG (*n* = 16)	MIX (*n* = 16)	*p* Values
Energy (kcals/kg)	24.9 ± 6.3	25.4 ± 7.2	24.4 ± 5.5	*p* = 0.66
Carbohydrates (g/kg)	3.4 ± 0.8	3.6 ± 0.9	3.1 ± 0.7	*p* = 0.15
Fat (g/kg)	0.9 ± 0.4	0.9 ± 0.5	0.9 ± 0.3	*p* = 0.91
Protein (g/kg)	1 ± 0.4	0.9 ± 0.2	1.1 ± 0.5	*p* = 0.31
Plant protein (g/kg) *	0.5 ± 0.2	0.6 ± 0.2	0.4 ± 0.1	*p* = 0.001
Animal protein (g/kg) *	0.5 ± 0.4	0.2 ± 0.2	0.7 ± 0.5	*p* = 0.001
Essential Amino Acids				
Isoleucine (g/kg) *	0.09 ± 0	0.1 ± 0	0.07 ± 0	*p* < 0.001
Leucine (g/kg) *	0.09 ± 0.1	0.07 ± 0	0.1 ± 0.1	*p* = 0.001
Valine (g/kg) *	0.08 ± 0.1	0.07 ± 0.1	0.08 ± 0	*p* = 0.001
Threonine (g/kg) *	0.05 ± 0	0.03 ± 0	0.06 ± 0	*p* = 0.001
Lysine (g/kg) *	0.07 ± 0.1	0.04 ± 0	0.1 ± 0.1	*p* < 0.001
Methionine (g/kg) *	0.03 ± 0	0.02 ± 0	0.05 ± 0.1	*p* < 0.001
Phenylalanine (g/kg) *	0.06 ± 0	0.04 ± 0	0.1 ± 0	*p* = 0.001
Histidine (g/kg) *	0.03 ± 0	0.02 ± 0	0.04 ± 0	*p* = 0.001
Tryptophan (g/kg) *	0.01 ± 0	0.01 ± 0	0.02 ± 0	*p* = 0.001

* = different between groups.

## Data Availability

Data are made available upon reasonable request.
